# At the genetic crossroads of *Leishmania*: Emerging hybrids reshaping disease patterns

**DOI:** 10.1371/journal.ppat.1013213

**Published:** 2025-06-03

**Authors:** Tiago R. Ferreira

**Affiliations:** Laboratory of Parasitic Diseases, National Institute of Allergy and Infectious Diseases, National Institutes of Health, Bethesda, Maryland, United States of America; Biology Centre, Academy of Sciences of the Czech Republic, CZECHIA

## Introduction

**Fig 1:**
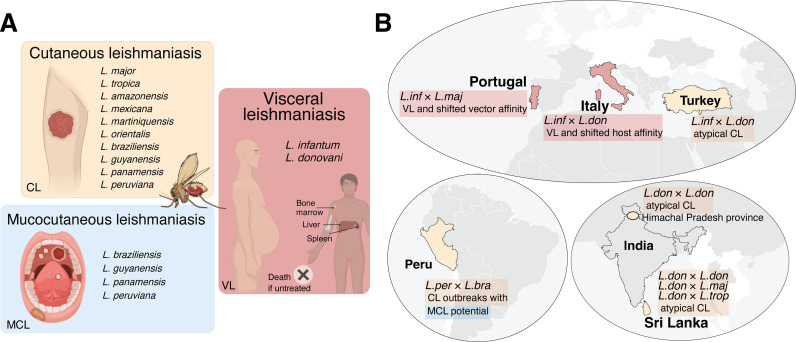
A. **Leishmaniasis is a suite of diseases with distinct clinical outcomes, ranging from cutaneous leishmaniasis (CL) and mucocutaneous leishmaniasis (MCL), to the deadly visceral leishmaniasis (VL). A non-exhaustive list of representative species typically associated with each form of leishmaniasis is presented.** *L.* (*Mundinia*) *martiniquensis* and *L.* (*Mundinia*) *orientalis* are likely transmitted by biting midges; all other species listed are transmitted by phlebotomine sand flies. **B**. Well-characterized examples of natural hybrids exhibiting epidemiological shifts are depicted in three different regions: South America, the Mediterranean, and South Asia. The geographical location, the parental genomes of each hybrid, and their associated changes of disease patterns are highlighted in different colors: yellow = CL, soft red = VL, blue = MCL, dark gray = other countries endemic for leishmaniasis. Species short names: *L.per* = *L. peruviana*, *L.bra* = *L. braziliensis*, *L.inf* = *L. infantum*, *L.maj* = *L. major*, *L.don* = *L. donovani*, *L.trop* = *L. tropica*. Illustration created using the NIAID NIH BIOART Source (bioart.niaid.nih.gov/bioart/458) and BioRender.com. Maps were created with the *rnaturalearth* R package, using shapefiles available at naturalearthdata.com/downloads/50m-cultural-vectors/.

Leishmaniasis is a devastating, neglected vector-borne disease that poses a significant threat to humans and domestic animals across 99 endemic countries. It is caused by protist parasites of the *Leishmania* genus, with an estimated 700,000–1 million new cases reported annually, including autochthonous cases in regions previously considered non-endemic [1]. Symptomatic leishmaniasis presents a broad spectrum of clinical outcomes, ranging from self-limiting skin lesions to severe facial disfigurement and, in the visceral form, even death (Fig 1A). The most widespread form of the disease is cutaneous leishmaniasis (CL), characterized by skin lesions of varying pathology [1,2]. In 5–10% of cutaneous cases in Latin America, with or without concurrent skin lesions, parasites affect the facial mucosa, leading to mucocutaneous leishmaniasis. Visceral leishmaniasis (VL) manifests as enlargement of the spleen and liver, anemia, and irregular bouts of fever. Without treatment, VL is fatal in over 95% of cases [1,2].

## Parasite genotype shapes leishmaniasis clinical outcome

While host immunity, co-infections (e.g., HIV), co-morbidities (e.g., malnutrition), and other factors contribute to disease manifestation, parasite genetics play a pivotal role in determining the pathogenesis of human leishmaniasis. Clinical outcomes in immunocompetent patients are mainly shaped by the species of *Leishmania* parasites usually transmitted through the bite of an infected female phlebotomine sand fly. More than 20 *Leishmania* species infect humans and are spread by over 90 different sand fly species worldwide [3]. The *Leishmania* species are currently classified into four subgenera: *Leishmania*, *Viannia*, *Mundinia*, and *Sauroleishmania*, with different geographic distribution, vector compatibility, and vertebrate host range. With the widespread availability of multilocus genotyping and whole-genome sequencing, recent studies have advanced our knowledge of parasite-disease associations. Among these findings, the emergence of genomic variants implicated in atypical disease dynamics (e.g., ‘visceral species’ causing cutaneous disease) suggests that strain genotype, rather than species identity *per se*, determines disease outcome in leishmaniasis. Investigating the universal drivers of genetic heterogeneity in these parasites is key to understanding their epidemiology, the emergence of variant pathogens causing atypical disease, the evolution of virulence, and the spread of drug resistance. This review explores how genetic exchange and hybridization may impact various aspects of leishmaniasis, with a focus on natural hybrids driving epidemiological shifts.

## Mechanisms of genetic exchange in *Leishmania*

Although traditionally regarded as predominantly clonal organisms (Predominant Clonal Evolution model discussed in detail in [4]), accumulating evidence demonstrates that *Leishmania* undergoes different types of genetic exchange, resulting in hybrid strains both in laboratory settings and natural populations [5,6]. *Leishmania* employs three major mechanisms of genetic exchange with distinct potential biological impacts and evolutionary significance. These mechanisms, so far demonstrated only in sand fly stages, are not mutually exclusive and may represent independent, extant processes in natural populations. Considering that climate change and human disturbances have led to the expansion of vector habitats [1], the risk of vertebrate host and sand fly co-infection by multiple strains/species may significantly increase. This could result in the rise of hybrid populations, leading to atypical variants of public health importance, with outbreak potential and new animal reservoir associations.

Meiotic sex represents a widespread and predominant mechanism of genetic exchange in eukaryotes. However, divergent single-celled organisms often exhibit cryptic sexual reproduction, in which meiosis and the fusion of sexual forms (syngamy) have not been directly observed. In contrast to other protist parasites, such as apicomplexans (e.g., *Plasmodium*), sex in kinetoplastids is cryptic, non-obligatory, and seems to constitute an alternative reproductive strategy within the vector [7]. In *Leishmania*, genetic and molecular evidence have revealed a meiosis-like recombination in sand flies, resulting in progeny that combines the genomes of both parents following a Mendelian segregation pattern [5,7]. Additionally, recently described self-mating (i.e., intraclonal sex) and/or backcrossing can further expand the potential for novel genetic traits [7,8].

Beyond classical meiosis, parasites can exchange genetic material through non-sexual means. Recent works have demonstrated that *Leishmania* parasites release DNA-loaded extracellular vesicles (EVs) [9], which can be taken up by other parasites, mediating horizontal gene transfer (HGT), through a process named ‘vesiduction’ [10]. HGT transfers discrete genetic elements such as drug resistance genes between parasites without forming full genomic hybrids [9,10]. EVs from drug-resistant *Leishmania* contain resistance gene amplicons that can be delivered to drug-sensitive parasites. Vesiduction may thus facilitate the dissemination of drug resistance in natural parasite populations.

Another mechanism, still poorly understood, is characterized by the generation of polyploid progeny (>2n), hypothetically arising from the fusion of diploid (2n) *Leishmania* or the fusion of haploid (1n) and diploid cells. This inferred non-meiotic hybridization has been described at low frequencies in experimental crosses within the sand fly [5,7] and is highly favored *in vitro* under conditions that induce DNA stress in the parasites [11]. Remarkably, these cells have not been described to undergo reduction of genomic content in experimental conditions. It is possible that this sexual-like mechanism emerged in ancient eukaryotes to provide undamaged chromosome copies, as a template for DNA repair. Although unequivocal evidence of natural genetic exchange in the wild has only been reported for meiosis-like hybridization, all three mechanisms mentioned above may contribute to the remarkable genomic plasticity and genetic variation observed in *Leishmania* parasites.

## Natural *Leishmania* hybrids associated with atypical epidemiology

Although sex appears to be facultative in *Leishmania*, several natural hybrid isolates have been reported across both ‘Old World’ and ‘New World’ species. Only those with potential relevance to the epidemiology and outcomes of leishmaniasis are discussed here (Fig 1B). Of note, the first natural hybrids between two highly divergent species, *Leishmania infantum* (VL agent) and *Leishmania major* (CL agent) were isolated from immunocompromised VL patients in Portugal in the late 1990s [12]. Although rare in the wild, an *L. infantum*–*L. major* cross raised concerns that genetic exchange might allow parasites to exploit new vectors or vertebrate hosts. Indeed, the two hybrids were experimentally demonstrated to generate mature infections in the midguts of an *L. major*-exclusive vector, *Phlebotomus papatasi*, which is not permissive to *L. infantum* strains [13]. In this case, genetic exchange enabled the expression of *L. major* cell surface lipophosphoglycans in a strain causing deadly VL, allowing it to survive in the specific vector. In principle, this could expand the potential geographical range of the parasite in nature.

Recent genomic analyses described a remarkable heterogeneity of *Leishmania* in isolates from northern Italy, grouping them with known hybrids from Cyprus and indicating an *L. infantum–Leishmania donovani* cross-lineage spanning these geographical areas. This *L. infantum*–*L. donovani* hybrid displays an unusual, exclusive affinity for human infection and has not been found in the typical *L. infantum* canine reservoirs to date [14]. It was suggested that the recent increase in human VL incidence in Italy may be linked to this emerging hybrid population in the region. This atypical vertebrate host affinity illustrates how hybridization may impact the zoonotic/anthroponotic transmission potential of leishmaniasis, fundamentally altering disease prevalence and risk factors.

A leishmaniasis outbreak in Turkey was the first report involving a natural hybrid with a non-classical association between parasite species identity and clinical presentation. A parasite lineage initially typed as *L. infantum* was the likely causative agent of hundreds of CL cases in the Çukurova region [15]. Whole-genome sequencing of parasite isolates from a patient and sand flies across two different areas in Çukurova revealed that these parasites originated from a single recent hybridization event between a local *L. infantum* strain and another strain part of the *L. donovani* complex [6]. This highly active leishmaniasis focus highlights the potential of a single hybridization event to impact parasite transmission potential and facilitate the emergence of a widespread outbreak.

In South Asia, human-derived *Leishmania* isolates from Sri Lanka provide an archetypal example of how genetic exchange can influence disease epidemiology on a national scale. Extensive hybridization and introgression events have played a role in shaping atypical disease patterns in the country [16]. Strikingly, CL in Sri Lanka is caused primarily by *L. donovani*, a prototypical VL species, as well as a recently described smaller population of endemic *Leishmania tropica* strains (classical CL species) [17]. The identification of unusually high genomic heterozygosity in *L. donovani* isolates, along with the presence of multiple cross-species hybrids between *L. donovani* and cutaneous *L. major* and *L. tropica*, indicates a link between genetic exchange and atypical cutaneous disease in Sri Lanka. Phylogenomic analyses suggest that both intra- and interspecies hybridization may explain why *L. donovani* infection on the island manifests as a skin-localized disease, in contrast to neighboring India, where phylogenetically distinct *L. donovani* causes deadly visceral disease. Notably, the sole exception in India is an intraspecies *L. donovani* hybrid that also causes CL in the Himachal Pradesh province without prior VL manifestation [18].

In South America, genetic exchange among *Leishmania* species, specifically within the *Viannia* subgenus, has led to frequent hybridization in areas where species coexist sympatrically. In the Peruvian Andes, *Leishmania peruviana* has long been the cause of mild cutaneous lesions, while *Leishmania braziliensis* can lead to mucocutaneous disease. However, *L. braziliensis*–*L. peruviana* hybrids have been associated with severe cutaneous disease outbreaks in Peru since the 1990s [19], with potential for mucosal metastasis. In experimental hamster infections, this hybrid lineage displayed increased cutaneous disease severity and aggressive late relapse when compared to either parental species [20]. Genetic analysis revealed a high copy number of the surface virulence factor GP63, which may be linked to the exacerbated virulence phenotype. Thus, recombination can generate *Leishmania* genotypes of increased virulence, capable of causing severe or unusual manifestations even in regions where typically milder species predominate.

## Challenges in vaccine development associated with genetic exchange

The genomic admixture of parental lineages introduces novel genetic variants into parasite populations, which can complicate confident diagnostics and/or immunization. No leishmaniasis vaccines are currently registered for human use, but several targeted vaccine candidates have been designed against often species-specific *Leishmania* antigens, assuming stable antigenic profiles [2]. Hybrid parasites, however, combine antigens from genetically distinct lineages, potentially creating antigenic mosaics that may escape immunity. This complexity requires vaccine designs incorporating broadly protective antigen targets conserved across multiple species or hybrid backgrounds. Approaches that favor pan-*Leishmania* formulations could neutralize diverse strains, including hybrids.

## Conclusion

In conclusion, the increasing detection of genetic exchange in *Leishmania* highlights the necessity of enhanced parasite genomic surveillance in highly endemic regions. Hybrids may inherit from both parental strains the ability to infect different vectors or persist in a specific vertebrate host tissue, effectively broadening their transmission potential. Overall, genetic exchange has the potential to reshape local leishmaniasis epidemiology, parasite virulence, transmission dynamics in endemic areas, host tissue tropism, and drug resistance, calling for adaptive control measures. Future efforts should prioritize mapping hybrid hotspots, identifying environmental factors driving recombination, and exploring the impact of rapid non-meiotic genetic exchange in the wild. Strengthening parasite surveillance and recognizing the importance of natural genetic exchange events will inform improved diagnostic tests and therapeutic strategies for targeted public health interventions to effectively control severe disease.
